# COX-2 Deficiency Promotes White Adipogenesis via PGE2-Mediated Paracrine Mechanism and Exacerbates Diet-Induced Obesity

**DOI:** 10.3390/cells11111819

**Published:** 2022-06-02

**Authors:** Chunqing Wang, Xing Zhang, Liping Luo, Yan Luo, Dandan Wu, Dianna Spilca, Que Le, Xin Yang, Katelyn Alvarez, William Curtis Hines, Xuexian O. Yang, Meilian Liu

**Affiliations:** 1 Department of Biochemistry and Molecular Biology, University of New Mexico Health Sciences Center, Albuquerque, NM 87131, USA; chuwang@salud.unm.edu (C.W.); xzhang1@salud.unm.edu (X.Z.); llp3253@163.com (L.L.); luoyan_2018@163.com (Y.L.); dspilca@unm.edu (D.S.); qle@unm.edu (Q.L.); xinyang@salud.unm.edu (X.Y.); kmalvarez@salud.unm.edu (K.A.); wchines@salud.unm.edu (W.C.H.); 2 Department of Molecular Genetics and Microbiology, University of New Mexico Health Sciences Center, Albuquerque, NM 87131, USA; dwu@salud.unm.edu (D.W.); xyang@salud.unm.edu (X.O.Y.); 3 Autophagy Inflammation and Metabolism Center for Biomedical Research Excellence, School of Medicine, University of New Mexico Health Sciences Center, Albuquerque, NM 87131, USA

**Keywords:** COX-2, PGE2, white adipogenesis, adipocyte hypertrophy, obesity, PPARγ

## Abstract

Cyclooxygenase-2 (COX-2) plays a critical role in regulating innate immunity and metabolism by producing prostaglandins (PGs) and other lipid mediators. However, the implication of adipose COX-2 in obesity remains largely unknown. Using adipocyte-specific COX-2 knockout (KO) mice, we showed that depleting COX-2 in adipocytes promoted white adipose tissue development accompanied with increased size and number of adipocytes and predisposed diet-induced adiposity, obesity, and insulin resistance. The increased size and number of adipocytes by COX-2 KO were reversed by the treatment of prostaglandin E2 (PGE2) but not PGI2 and PGD2 during adipocyte differentiation. PGE2 suppresses PPARγ expression through the PKA pathway at the early phase of adipogenesis, and treatment of PGE2 or PKA activator isoproterenol diminished the increased lipid droplets in size and number in COX-2 KO primary adipocytes. Administration of PGE2 attenuated increased fat mass and fat percentage in COX-2 deficient mice. Taken together, our study demonstrated the suppressing effect of adipocyte COX-2 on adipogenesis and reveals that COX-2 restrains adipose tissue expansion via the PGE2-mediated paracrine mechanism and prevents the development of obesity and related metabolic disorders.

## 1. Introduction

Obesity, a disorder characterized by excess adiposity, has become a primary cause of insulin resistance, type 2 diabetes, and cardiovascular diseases. Pathological expansion of adipose tissue (AT) is accompanied by adipose hypoxia, angiogenesis, remodeling, and inflammation, thereby causing systemic insulin resistance and type 2 diabetes [1,2]. However, the mechanisms regulating adipocyte differentiation, maturation, and turn over are incompletely understood.

The nuclear hormone receptor peroxisome proliferator-activated receptor gamma (PPARγ) is a mast regulator of adipocyte differentiation [3,4]. Upon its activation by binding to its endogenous ligands or synthetic ligands such as thiazolidinediones (TZDs), PPARγ promotes adipogenesis and insulin sensitivity [3,4]. It is primarily expressed in adipocytes [4] and present in other cell types such as regulatory T cells with a lesser extent [5,6]. Activated PPARγ binds to its responsive elements in the promoter and induces adipogenesis- and lipogenesis-related genes, including *Cd36*, lipoprotein lipase (*Lpl*), fatty acid-binding protein (*aP2*), CCAAT/enhancer-binding protein alpha (*C/ebpα*) and other adipogenesis markers such as adiponectin [4,7,8]. In addition to ligand stimulation, PPARγ transcriptional activity is controlled by its protein modification, including phosphorylation (Ser273) [9,10,11], acetylation (Lys268 and Lys293) [12], sumoylation [13,14,15,16,17], glycosylation [18] and ubiquitination [19,20,21,22]. Such modifications of PPARγ are critical for its action, stability, and selective targeting and are functionally connected [9,10,11,12,19,20]. For instance, ligand binding not only increases the transcriptional function of PPARγ but also induces its ubiquitination and subsequent degradation by the proteasome [19]. Sirt1-mediated deacetylation of PPARγ at Lys268 and Lys293 is critical for its stabilization and has been linked to its adipogenesis-inducing effect (unpresented data in the referred study) [12]. Additional factors, including interferon γ (IFNγ) and tumor necrosis factor alpha (TNFα), have been shown to regulate PPARγ degradation via a proteasome-dependent mechanism in adipocytes [23,24,25], indicative of the significance of PPARγ stabilization in its function. However, while fatty acid metabolites are considered potential endogenous ligands of PPARγ, the mechanisms underlying PPARγ regulation are incompletely understood.

Cyclooxygenase (COX), a rate-limiting enzyme responsible for the biosynthesis of prostaglandins (PGs), exists in three isoforms; COX-1, the constitutive form, COX-2, the inducible form, and COX-3, the splicing variant [26]. COX-2 oxygenates arachidonic acid and converts it into a number of PGs, including PGD2, PGE2, PGF2α, and prostacyclin (PGI2), all of which exert diverse hormone-like effects via autocrine or paracrine mechanisms [26]. The accumulated evidence shows that PGs modulate adipogenesis by acting as an agonist or analog of Peroxisome Proliferator-Activated Receptor γ (PPARγ) [27]. It has also been suggested that the COX-2/PG axis plays a critical role in regulating AT inflammation and obesity-induced insulin resistance [28,29,30,31,32,33]. In addition, the COX-2/PG pathway has been shown to promote thermogenic programming and white adipose tissue (WAT) browning, although it is still controversial whether COX-2 is induced by cold and is correlated with obesity [28,34,35,36,37]. On the other hand, while COX-2 overexpression in adipocytes leads to protection against diet-induced obesity and inflammation, and COX-2 in adipocytes mediates the anti-inflammatory benefits of intermittent fasting [38,39], whether endogenous COX-2 in adipocytes is implicated in adiposity and obesity is unclear.

We recently showed that adipose COX-2 is inversely correlated with obesity in humans and in rodents, and that intermittent fasting (IF) restores the expression level of COX-2 in adipose tissue of obese mice [39]. In addition, COX-2 in adipocytes mediates the anti-inflammatory effects of intermittent fasting in adipose tissue despite similar anti-obesity effects of IF in adipocyte-specific COX-2 KO and control mice [39]. In the present study, we specifically characterized the role of adipose COX-2 in regulating adipogenesis and found that COX-2 in adipocytes exerts an anti-obesity effect by suppressing white adipogenesis. PGE2 but not PGI2 and PGD2 attenuates the increased adipogenesis of COX-2 KO primary stromal cells via PKA-mediated suppression of PPARγ function. Moreover, adipocyte-specific COX-2 deficient mice are prone to diet-induced obesity and insulin resistance which was ameliorated by PGE2 treatment. Taken together, our study reveals that COX-2 in adipocytes plays a critical role in the control of adipocyte differentiation by the PGE2-mediated paracrine mechanism.

## 2. Materials and Methods

### 2.1. Materials

Antibodies against COX-2 (12282), PPARγ (2443), PGC1α (2178), and PKA (9621) were from Cell Signaling Technology (Danvers, MA, USA). Polyclonal antibodies to adiponectin and anti-tubulin were kindly provided by Drs. Lily Dong and Feng Liu at UT Health San Antonio (San Antonio, TX, USA) as described previously [40]. The anti-UCP1 (ab23841) and anti-Plin1 (ab61682) were purchased from Abcam (Discovery Drive, Cambridge, CB2 0AX, UK). BODIPY 493/503 (D3922) and DAPI (D3571) were from Thermo Fisher Scientific (Waltham, MA, USA). PGD2 (41598-07-6), PGE2 (14010), PGI2 (69552-46-1), L-161,982 (10011565), TG6-10-1 (23444), SC-51089 (10011561), and Rosiglitazone (71740) were obtained from Cayman Chemical Company (Ann Arbor, MI, USA). KT5720 (K3761) and Isoproterenol (I6504) were from Sigma-Aldrich (St. Louis, MO, USA). H89 (371963) was purchased from Millipore (Burlington, MA, USA).

### 2.2. Animals

The COX-2 floxed mice were generously provided by Dr. Harvey R. Herschman at the University of California (Los Angeles, CA, USA). Adipocyte-COX-2 specific knockout (KO) mice were generated by crossing COX-2 floxed mice with adiponectin cre mice (Jackson Laboratory, Bar Harbor, ME, USA. Stock number 10803). COX-2 floxed littermates were used as controls. The knockout efficiency was confirmed in adipose tissue and other tissues by Western blot analysis and Real-Time PCR shown in our previous work. Unless otherwise noted, male mice were used for all experiments. Animals were housed in a specific pathogen-free barrier facility with a 12-h light/12-h dark cycle with free access to food and water. For the high-fat diet challenge study, 6-week-old mice were fed with a high fat diet (HFD) (45% kcal fat) from Research Diets Inc. (D12451; New Brunswick, NJ, USA) for 8 or 16 weeks unless otherwise specified, and normal chow diet (NCD) provided by the animal facility at the UNMHSC. All animal experimental protocols were reviewed and approved by the Animal Care Committee of the University of New Mexico Health Sciences Center.

### 2.3. Administration of PGE2

PGE2 was dissolved in 100% dimethyl sulfoxide (DMSO) and diluted in 0.9% Sodium Chloride to reach the concentration of 5 µg/mL. Four-month-old male COX-2 KO, COX-2 floxed, or wild type C57BL/6 mice were administered with PGE2 or vehicle through intraperitoneal injection at 50 µg/kg every other day for 2 weeks.

### 2.4. Primary Culture and Differentiation of Adipocytes

Primary stromal vascular fractions (SVFs) from iWAT of 3-week-old COX-2 knockout and control mice were isolated, cultured, and differentiated into adipocytes according to the procedure as described previously [41]. In brief, the confluent stromal cells were cultured in the induction medium (DMEM with 10% Fetal Bovine Serum, plus 8 μg/mL insulin, 0.1 μg/mL Dexamethesine, and 112 μg/mL Isobutylmethylxanthine) for 4 days followed with differentiation medium (DMEM with 10% Fetal Bovine Serum, plus 8 μg/mL insulin) for 2 days, and treated with or without PGD2, PGE2 or PGI2 for 6 days during the differentiation. On day 6, cells were harvested for Western blot analysis, Real-Time PCR, or staining. Alternatively, day-6 differentiated primary COX-2 KO and control adipocytes were cultured with a fresh differentiation medium for 24 h. The media was collected and used to treat wild-type primary preadipocytes isolated from inguinal fat that underwent differentiation to adipocytes. This treatment was started on day 1 and lasted for 6 days during differentiation.

### 2.5. Treatment of PGE2 Receptor Antagonists or PKA Activator/Inhibitors during Differentiation

Primary stromal cells isolated from inguinal fat of COX-2 KO and control mice were differentiated into adipocytes. Starting from day 1 of differentiation, cells were treated with β-adrenoceptor agonist isoproterenol (10 μM) for 6 days. For the PGE2 study, primary stromal cells from C57BL/6 mice were differentiated and treated with PGE2 with cotreatment of EP1 antagonist SC-51089 (1 μM), EP2 antagonist TG6-10-1 (1 μM), EP4 antagonist L-161,982 (1 μM), or PKA specific inhibitor KT5720 (10 μM) or H89 (10 μM) for 6 days from day 1 of differentiation.

### 2.6. Hematoxylin and Eosin (H&E) and Oil Red O Staining

For the H&E staining, tissues were fixed with 10% formalin for 24 h and embedded in paraffin. Tissue sections (6-μm thick) were stained with H&E according to standard protocols and analyzed using the NIH Image J software (Bethesda, MD, USA). To analyze the adipocyte size in adipose tissue, stained tissues were visualized with NanoZoomer Slide Scanner (Leica Biosystems, Buffalo Grove, IL, USA). Pick eight fields per animal genotype were analyzed and each field randomly count 30 cells’ size by Image J. For the quantification of total adipocyte numbers in adipose tissue, it was estimated by manual counting of cells on H&E slides from at least three 20× fields of three mice per genotype and approximated assuming cubic packing as a previous study described [42]. Adipocyte cell diameters of adipose tissue were measured to determine the mean adipocyte diameter. The mean adipocyte density (cells/unit volume) was next calculated from the mean adipocyte diameter assuming cubic closest packing of adipocytes. The total number of adipocytes in each epididymal fat pad during normal feeding or HFD feeding was then calculated from the density of adipocytes (cells/unit volume) and the measured total volume of each epididymal fat pad. For the Oil Red O staining, differentiated primary adipocytes were fixed with 4% formalin for 10 min and stained with Oil Red O in 60% isopropanol for 20 min [43], and images were taken by EVOS FL Cell Imaging System.

### 2.7. Glucose Tolerance Test (GTT) and Insulin Tolerance Test (ITT)

For the GTT, mice were fasted for 12 h, and fasted glucose was measured using a Glucometer (GE, Boston, MA, USA) by tail bleeds. Then mice were intraperitoneally injected with 2 g glucose/kg of body weight, and blood glucose was measured at the time of 15 min, 30 min, 60 min, and 120 min after glucose injection. For the ITT, mice were fasted for 4 h, and fasted glucose was measured using a Glucometer (GE) by tail bleeds. Then mice were intraperitoneally injected with 0.75(U/mL) insulin/kg of body weight, and blood glucose was measured at the time of 15 min, 30 min, 60 min, and 90 min after insulin injection.

### 2.8. DEXA Scanning

To check the body composition, mice were anesthetized through intraperitoneal injection with 0.1 mL/10 g animal body weight Ketamine/Xylazine (10 mg/mL Ketamine and 1 mg/mL Xylazine). Bone mineral density, lean mass, fat mass, total body weight, and fat percentage were determined by dual-energy X-ray absorptiometry (DEXA) (GE Medical Systems, Madison, WI, USA).

### 2.9. Immunofluorescence Staining

Adipocytes in 24-well plates were fixed in 4% paraformaldehyde for 10 min, and then permeabilized with 0.1% saponin in 3% bovine serum albumin (BSA) for 30 min. Incubation with anti-Plin1 for an hour and followed by secondary antibodies as well as anti-Bodipy together for half an hour. Cell Nuclei were stained with DAPI (blue). Confocal images were taken using Cellomics Image System (Thermo Fisher Scientific) as previously described [44].

### 2.10. Real-Time PCR and Western Blot

Total RNA was extracted from mice tissues or primary cultural cells using the RNeasy Lipid Tissue Mini Kit (Qiagen, Germantown, MD, USA). The purity and concentration of total RNA were determined by a NanoDrop spectrophotometer (Thermo Fisher Scientific). One μg of total RNA was reverse-transcribed using a cDNA kit (AB Applied Biosystems, Waltham, MA, USA). Real-time PCR amplification was detected using SYBR Green PCR master mixture (Qiagen) on a Roche 480 Real-time PCR system (Basel, Switzerland). The primer sequences used in real-time PCR are shown in Supplementary Table S1. Western blot analysis was performed following the procedures described previously [45].

### 2.11. Statistics

Statistical analysis of the data was performed using a two-tailed Student’s *t*-test between two groups or one-way ANOVA among three different groups. All the results were presented as the mean ± S.E.M., and a *p*-value of <0.05 was considered to be statistically significant.

## 3. Results

### 3.1. Adipocyte-Specific Depletion of COX-2 Predisposes Diet-Induced Adiposity and Insulin Resistance

**Figure 1:**
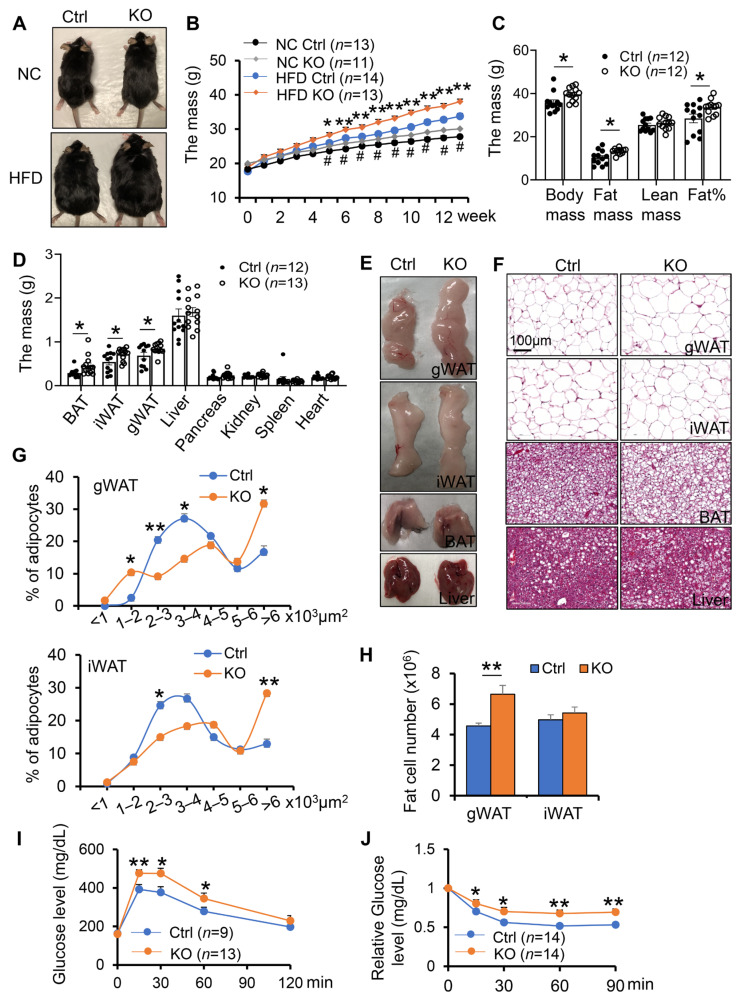
Adipocyte-specific depletion of COX-2 predisposes diet-induced obesity and insulin resistance.6-week-old male COX-2 KO and control mice were fed with a normal chow diet (NCD) or high-fat diet (HFD) for 12 weeks, and used for the following studies. (**A**). Representative images of COX-2 KO and control mice fed with NCD or HFD for 12 weeks. (**B**). COX-2 KO mice gained more weight than control littermates during growth under NCD conditions, and were more sensitive to HFD-induced obesity. Body weight was measured weekly prior to and after mice were fed with NCD or HFD. (**C**). COX-2 deficiency led to increased fat mass and fat percentage compared to control littermates under HFD conditions. The lean mass, fat mass, total mass, and fat percentage of HFD mice were measured using DEXA scanning. (**D**). The mass of gWAT, iWAT, and BAT fat pads were significantly increased in HFD KO mice compared with controls. The mass of organs and fat pads were weighed after mice were euthanized. (**E**). Representative images of gWAT, iWAT, BAT, and liver in HFD KO and control mice. (**F**). H&E staining of gWAT, iWAT, BAT, and liver in HFD KO and control mice. Quantification of adipocyte size (**G**) and number (**H**) in gWAT and iWAT of COX-2 KO and control mice fed with HFD. COX-2 KO mice were more intolerant of glucose (**I**) and insulin (**J**) than control mice under HFD conditions. Data are presented as the mean ± SEM. T-Test was used for the analysis in Figure 1B–D,G,H, and ANOVA was used for the analysis of Figure 1I,J. * *p* < 0.05, ** *p* < 0.01, HFD control vs. HFD KO; # *p* < 0.05, NCD control vs. NCD KO.

COX-2 has been shown to play an important role in regulating inflammation and energy homeostasis [28,35,46,47]. However, little is known about the physiological role of COX-2 in adipocytes. To this end, we generated adipocyte-specific COX-2 knockout (KO) mice by crossing COX-2 floxed mice and adiponectin cre mice [39]. To investigate the potential effect of COX-2 on diet-induced obesity and insulin resistance in vivo, we fed the COX-2 KO mice and control littermates with HFD for 16-weeks. No significant difference in food intake was observed between COX-2 KO mice and their control mice during HFD feeding (Figure S1A). However, the differences in body size and weight were more pronounced between HFD-fed COX-2 KO mice and the control littermates (Figure 1A,B). The fat mass and fat percentage of COX-2 KO mice were also significantly greater compared with control littermates on HFD (Figure 1C–E). Consistent with this finding, the HFD-fed COX-2 KO mice showed larger fat cell size and increased fat cell number in gonadal white adipose tissue (gWAT) and inguinal WAT (iWAT) as well as severe hepatosteatosis compared with HFD-fed wild-type littermates (Figure 1F–H), leading to severe glucose and insulin intolerance after 8 weeks of HFD feeding (Figure 1I,J). These results suggest that COX-2 KO exacerbates diet-induced obesity and insulin resistance.

COX-2 deficiency had little effect on the food intake during HFD feeding (Figure S1A). Surprisingly, the decreased insulin tolerance via COX-2 deficiency was not pronounced despite a dramatic increase in body weight gain under 16 weeks of HFD feedings (Figure S1B,C), implying a distinct effect of adipocyte COX-2 on insulin sensitivity in the early and late stages of obesity. The anti-obesity property of COX-2 was restricted to males given the modest effects of COX-2 KO on body weight and insulin sensitivity in female mice (Figure S1D–F). Despite little effect on energy expenditure, food intake, activity, and cold-induced thermogenic gene expression under normal chow diet conditions (Figure S2), adipocyte-specific depletion of COX-2 reduced the basal expression of UCP1 and PGC1α (Figure S2D,F). In addition, COX-2 deficiency in adipocytes improves anti-inflammatory response under NCD conditions, while this improving effect was not observed post-16-week HFD feeding (Figure S3).

### 3.2. COX-2 Deficiency Enhances White Adipogenesis

**Figure 2:**
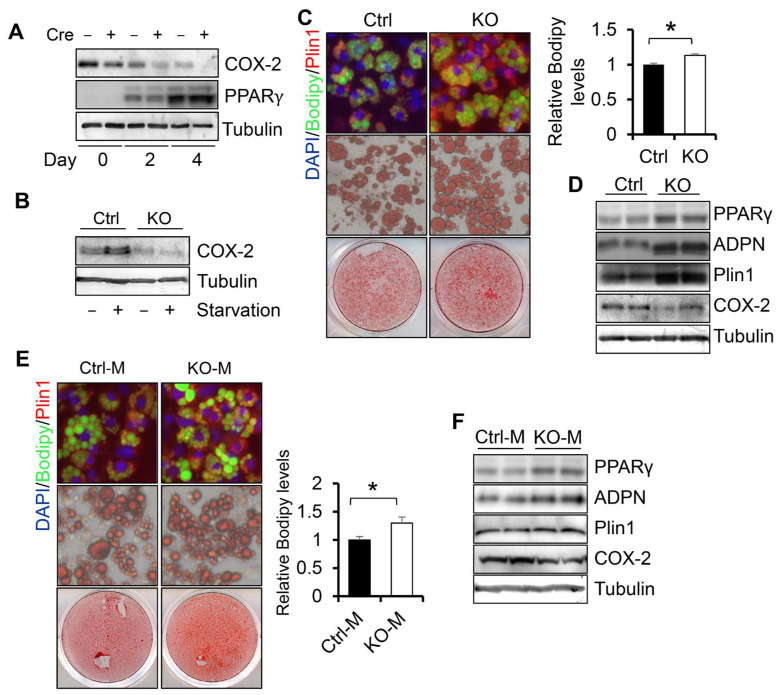
COX-2 deficiency enhances white adipogenesis. The stromal vascular fractions were isolated from white adipose tissue, cultured, and differentiated into adipocytes. (**A**). The expression levels of COX-2 and PPARγ in COX-2 KO and control cells during differentiation. (**B**). COX-2 was induced by starvation in control adipocytes but not in COX-2 KO cells. (**C**). COX-2 depletion enhanced white adipogenesis as presented by fluorescence staining of Bodipy, Plin1, and DAPI, and Oil red O staining in primary cells. On day 6, differentiated COX-2 KO and control cells were stained with antibodies of Bodipy and Plin1 or Oil red O. The relative levels of Bodipy were quantified. (**D**). Expression levels of adipogenic markers PPARγ, adiponectin, and Plin1 were significantly induced by COX-2 deficiency in day 6-differentiated primary adipocytes. (**E**). The media from primary COX-2 KO adipocytes increased the ratio of differentiation in control cells as presented by Oil red O staining and fluorescence staining of DAPI, Bodipy, and Plin1 in primary cells and quantitative of the relative Bodipy levels. (**F**). Expression levels of adipogenic markers PPARγ, adiponectin, and Plin1 were slightly induced by the treatment of COX-2 deficiency media in day 6-differentiated primary adipocytes. Figure 2A–F are the representative data from three independent experiments. T-Test was used for the analysis in Figure 2C,E. * *p* < 0.05.

To gain insight into the role of COX-2 in regulating adipogenesis, we performed differentiation of COX-2 KO primary preadipocytes. COX-2 protein levels were markedly decreased in COX-2 KO adipocytes compared to control cells during adipogenesis and upon starvation treatment (Figure 2A,B), indicating that COX-2 was successfully suppressed by COX-2 KO in adipocytes. The protein levels of COX-2 were suppressed during the differentiation and were notably induced by starvation in primary adipocytes (Figure 2A,B), suggesting a potential role of COX-2 in adipogenesis. Fluorescence and Red Oil O staining results showed that COX-2 deficiency promoted adipocyte differentiation indicated by the increased staining of lipid droplet marker Bodipy and upregulated expression levels of adipogenesis markers PPARγ and Plin1 (Figure 2C,D). Consistent with this, the media from COX-2 KO adipocytes enhanced adipocyte differentiation compared to the media from the control cells (Figure 2E,F), indicative of an autocrine or paracrine mechanism mediating the suppressing effect of COX-2 on adipocyte differentiation.

### 3.3. Depletion of COX-2 Promotes Adipocyte Maturation via PGE2-Mediated Paracrine Mechanism

**Figure 3:**
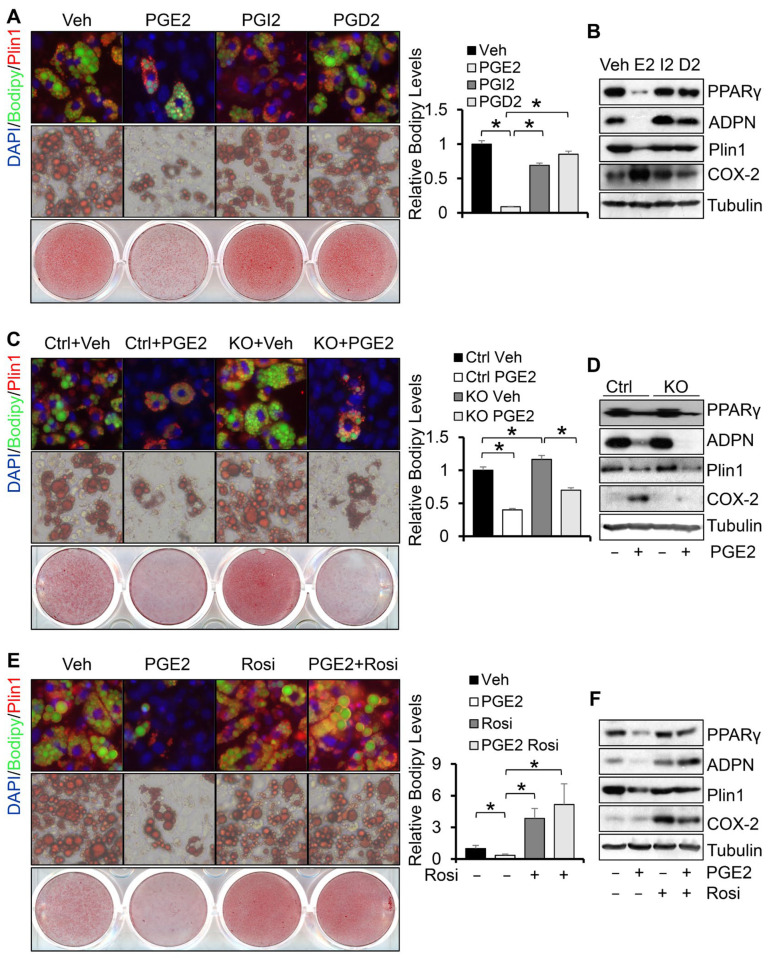
Depletion of COX-2 promotes adipocyte maturation via the PGE2-mediated paracrine mechanism. Treatment of 1 µM PGE2 but not PGD2 and PGI2 suppressed white adipogenesis presented by Oil red O staining and fluorescence staining of Bodipy and Plin1 (**A**) as well as the expression levels of adipogenic markers PPARγ, adiponectin, and Plin1 (**B**). Starting from differentiation, primary preadipocytes were treated with 1 µM PGE2, PGI2, and PGD2 for 6 days. Treatment of 1 µM PGE2 reversed COX-2 deficiency-induced white adipogenesis presented by Oil red O staining and fluorescence staining of Bodipy and Plin1 (**C**) as well as the expression levels of adipogenic markers PPARγ, adiponectin, and Plin1 (**D**) in primary adipocytes. Treatment of 10 µM PPARγ agonist Rosiglitazone restored PGE2 treatment-suppressed white adipogenesis presented by Oil red O staining and fluorescence staining of Bodipy and Plin1 (**E**) as well as the expression levels of adipogenic markers PPARγ, adiponectin, and Plin1 (**F**) in primary adipocytes. Data in Figure 3A,C,E are presented with means ± S.E.M. * *p* < 0.05.

To delineate the role of PGs in COX-2 deficiency-induced differentiation of white adipocytes, we treated primary adipocytes with PGD2, PGE2, or PGI2 during white differentiation. We found that treatment of PGE2 but not PGD2 or PGI2 dramatically suppressed the formation of lipid droplets and expression of adipocyte markers PPARγ and Plin1 (Figure 3A,B). In addition, PGE2 treatment reversed the increase in lipid droplets and expression of PPARγ, Plin1, and adiponectin in COX-2 KO adipocytes during differentiation (Figure 3C,D and Figure S4A,B). In agreement with these results, treatment of cells with PPARγ agonist rosiglitazone promoted the differentiation of primary adipocytes and diminished the suppressing effect of PGE2 on adipogenesis (Figure 3E,F), suggesting that COX-2 in adipocytes suppresses white adipogenesis via PGE2-mediated paracrine mechanism.

### 3.4. PGE2 Suppresses PPARγ Expression and Adipogenesis through PKA Signaling

**Figure 4:**
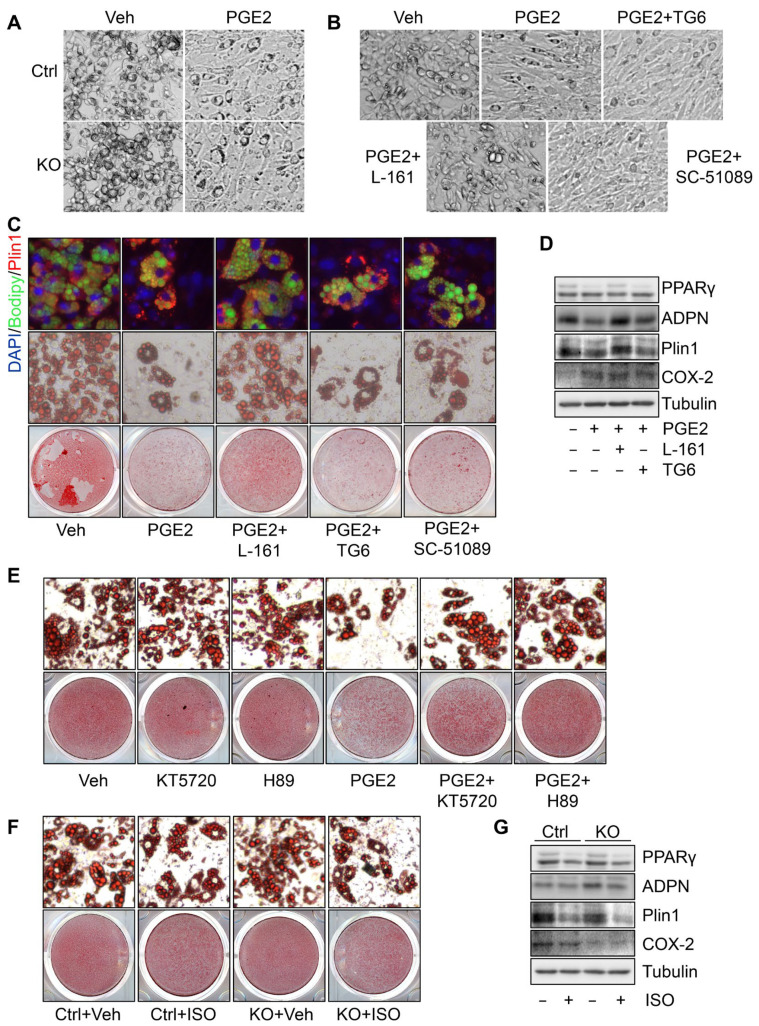
PGE2 suppresses PPARγ expression and adipogenesis through PKA signaling. (**A**). The treatment of PGE2 blocked the morphology alteration at the early stage of differentiation. Starting from differentiation, primary preadipocytes were treated with 1 µM PGE2, and the bright field images were taken on day two of differentiation. The suppressing effect of PGE2 on the white adipogenesis was restored by the treatment of PGE2 receptor EP4 antagonist L161,982 but not the antagonists of EP1 and EP2, SC-51089 and TG6-10-1 respectively, indicated by the bright field images of day two cells in differentiation (**B**), Oil red O/fluorescence staining (**C**), and expression levels of adipogenic markers PPARγ, adiponectin, and Plin1 (**D**). (**E**). Inhibition of PKA with its specific inhibitors KT5720 or H89 restored the suppressing effect of PGE2 on adipogenesis. Activation of PKA with isoproterenol suppressed COX-2 deficiency-induced white adipogenesis presented by Oil red O staining (**F**) and expression of PPARγ, adiponectin, and Plin1 (**G**). Figure 4A–G is the representative data from three independent experiments.

To dissect the role of PGE2 in COX-2 suppression of adipogenesis, we treated COX-2 KO primary cells at various phases of differentiation. We found that the treatment of PGE2 in early phase (day 1) but not late phase of differentiation (day 4) reversed COX-2 deficiency-induced adipogenesis (Figure 4A and data not shown). Consistent with this, blocking the signaling of PGE2 receptor EP4 but not EP1 and EP2 by treatment with their antagonists L161,982, SC-51089, and TG6-10-1, respectively, restored the suppressed adipogenesis by PGE2, presented by the morphology alteration of cells at the initiation of adipogenesis (Figure 4B) and lipid droplet and the expression of adipogenic markers PPARγ and adiponectin on day 6 of differentiation (Figure 4C,D). Given that PKA has been suggested as an intracellular effector of PGE2 [36] (Figure S4C), preadipocytes were treated with PGE2 and cotreated with PKA inhibitors KT5720 and H89 starting from the beginning of differentiation (Figure 4E). Inhibiting PKA signaling attenuated the suppressing effect of PGE2 on white adipogenesis indicated by Oil red O staining (Figure 4E) and immunofluorescent staining (Figure S4D). In contrast, activating PKA signaling by isoproterenol diminished the inducing effect of COX-2 deficiency on adipogenesis in primary cells (Figure 4F,G, and Figure S4E). These results together suggest that the suppressing effect of the COX-2/PGE2 axis on adipogenesis is mediated by PKA signaling.

### 3.5. Administration of PGE2 Reversed COX-2 KO-Induced White Adipogenesis In Vivo

**Figure 5:**
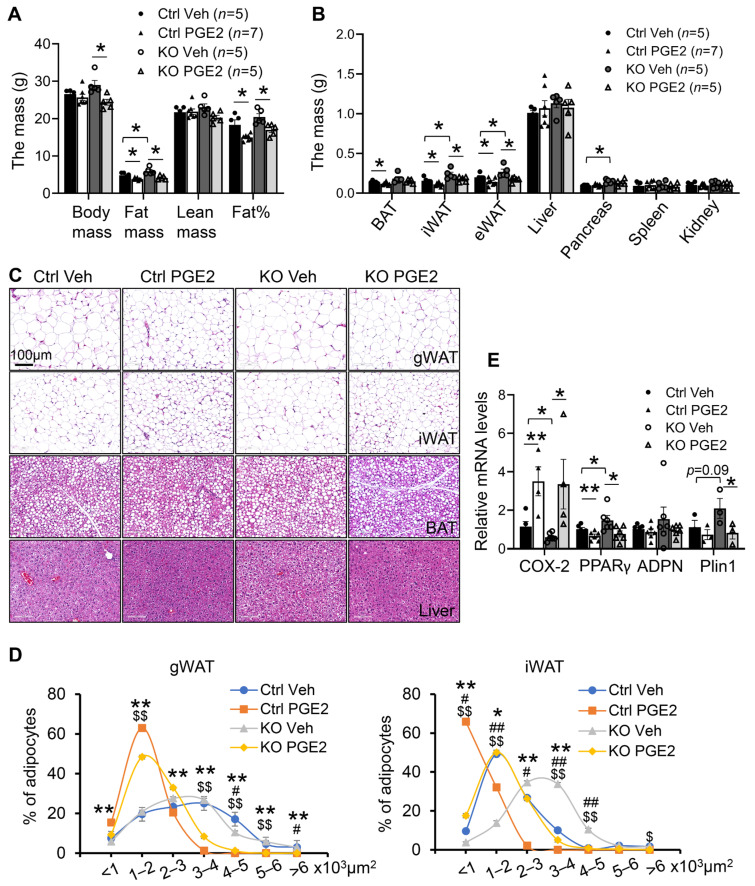
Administration of PGE2 reversed COX-2 KO-induced white adipogenesis in vivo. Six-month-old COX-2 KO mice were injected with PGE2 or vehicle for two weeks. (**A**). PGE2 administration significantly decreased body mass, fat mass, and fat percentage of COX-2 KO mice. *n* = 5–7/group. (**B**). Treatment of PGE2 suppressed the mass of three fat pads, inguinal, gonadal, and brown fat, and pancreas with no significant effect on liver, spleen, and kidney. (**C**). PGE2 administration decreased the size of adipocytes in gWAT and iWAT despite a little effect on BAT and liver in COX-2 KO mice. (**D**). Quantification of adipocyte size in figure (**C**) showing that a shift of large to small adipocytes by PGE2 treatment. (**E**). PGE2 administration suppressed the expression of PPARγ, adiponectin, and Plin1, although the reduction of adiponectin by PGE2 did not reach significance in COX-2 KO mice. Data in Figure 5A,B,E are presented with means ± S.E.M. * *p* < 0.05, ** *p* < 0.01. Data in Figure 5D are presented with means ± S.E.M. * *p* < 0.05 and ** *p* < 0.01 for Ctrl Veh vs. Ctrl PGE2; # *p* < 0.05 and ## *p* < 0.01 for Ctrl Veh vs. KO Veh; $ *p* < 0.05 and $$ *p* < 0.01 for KO Veh vs. KO PGE2.

To investigate the role of PGE2 in mediating the anti-obesity effect of COX-2 in adipocytes, we administered PGE2 into 4-month-old COX-2 KO and control mice. Consistent with our in vitro data (Figure 3 and Figure 4), administration of 50 µg/kg PGE2 by IP injection for 2 weeks reversed increased fat mass and fat percentage in gWAT and iWAT of COX-2 KO mice (Figure 5A,B). In support of this, PGE2 administration also significantly rescued adipocyte size in gWAT and iWAT of COX-2 KO mice (Figure 5C,D). Along this line, the upregulation of adipogenic markers PPARγ by COX-2 was diminished by PGE2 treatment (Figure 5E). These results were also supported by our recent work showing that PGE2 exerts a strong anti-obesity effect and improves COX-2-predisposed obesity under HFD conditions [39], indicating that the adipocyte COX-2/PGE2 axis is a negative regulator of white adipogenesis.

## 4. Discussion

Cyclooxygenase-2 (COX-2) is an integral component of inflammation, promoting an immune response to infection and injury by producing arachidonic acid-derived prostaglandins (PGs) and other lipokines in immune cells [48]. Interestingly, COX-2 is also expressed in adipocytes, converts arachidonic acid to a variety of PGs in response to nutritional stress, and mediates the metabolic benefits elicited by intermittent fasting (IF) [31,39]. However, the implication of adipose COX-2 in obesity is incompletely understood. Our study showed that COX-2 in adipocytes limits whit adipogenesis and suppresses pathological expansion of adipose tissue, thereby exerting an anti-obesity property. In addition, COX-2 in adipocyte suppresses progenitor cells’ commitment to differentiation via PGE2-mediated paracrine mechanisms that require PGE2 receptor EP4 and downstream PKA signaling.

COX-2 expression is restricted under basal conditions in adipocytes [31,37], and is markedly induced by a variety of physiological conditions such as fasting, adrenoceptor activation, and mTORC1 inhibition in adipocytes [30,31,36,39,44]. Along this line, COX-2 expression is tightly associated with lipolysis, a cellular process controlled by adrenergic signaling in adipocytes [28,31,49]. Additionally, IF promotes the production and release of COX-2 products PGs from adipocytes, including PGE2, PGD2, and PGI2, all of which communicate directly with progenitors and promote beige adipogenesis in the early phase [36,39]. COX-2 in adipocytes mediates IF-induced anti-inflammatory effect and -improved insulin sensitivity despite no significant difference in IF-elicited anti-obesity effect [39]. Given that COX-2 expression in adipose tissue is also suppressed by obesity in humans and in rodents [39], the present study addresses the anti-obesity effect of COX-2 in adipocytes and demonstrates that adipocyte-derived PGE2 inhibits progenitor cell differentiation into white adipocyte and prevents the development of adiposity and obesity. Consistent with this, overexpressing COX-2 in adipocytes prevents the development of obesity and adipose tissue inflammation [38]. In addition, the anti-obesity effect of COX-2 in adipocytes is selective in male mice given that adipocyte COX-2 deficiency has no significant effect on diet-induced body weight gain and insulin resistance in female mice (Figure S1). These results support that distinct from male mice, female mice are genetically protected against diet-induced insulin resistance as showed in other models, again suggesting the protective effect of the sex hormone estrogen in female mice [50,51,52].

Enzyme immunoassay (ELISA) and Mass Spec analyses have been widely used to assess the production and release of a variety of PGs, including PGE2, PGI2, and PGD2, in adipocytes [30,31,53]. Of note, the short half-lives of PGE2 and PGI2 are approximately 5 min and 10 min, respectively, as reported [54,55,56,57]. The metabolites of PGE2 such as 15-keto-13,14 dihydro-PGE_2_ appear to be more stable than PGE2 itself [56]. PGE2 secretion levels were about 50% lower in COX-2 KO adipocytes compared to the controls in 2-hr conditional media, and a similar effect was found in the 4-hr conditional media despite no significant difference [39]. The reducing effect of COX-2 KO on PGE2 secretion was retained in the 8- and 18-hr media, albeit to a lesser extent [39], indicative of the short half-life of adipocyte-derived PGE2 as well. The dynamic contribution of adipocytes vs stromal vascular cells to the total levels of PGs in adipose tissue microenvironment remains to be explored under various pathophysiological conditions. As the beneficial effects of COX-2 in adipocytes are abrogated under the later stage of obesity, it is possible that stromal vascular cells become a dominant source of PGs under such conditions. In agreement with this, the expression levels of COX-2 declined during adipogenesis (Figure 2), and the protective effect of COX-2 on adipose tissue inflammation only occurred at the early stage of obesity (Figure S3). Of note, PGE2 is a product of COX-2, while feedforward activates the *Cox-2* gene in primary adipocytes, likely via an autocrine or paracrine mechanism (Figure 2). PGE2-mediated feedforward mechanism magnifies nutritional stress-induced COX-2 expression and prostaglandin production in adipocytes. It is possible that PGE2 binds to its own receptor EP4 in this case through which activates PKA signaling and substantial *Cox-2* gene. This also suggests that PGE2-mediated feed-forward regulation of COX-2 may have an important impact on physiology, considering that the basal expression level of COX-2 is low, whereas the expression level can be markedly upregulated under certain circumstances such as fasting or mTORC1 inhibition conditions.

The mechanisms underlying the anti-obesity effect of COX-2 are complicated but likely mediated by its product’s PGs-mediated paracrine mechanisms. The media collected from COX-2 KO adipocytes contains lower levels of PGE2 and PGI2 with no significant effect on PGD2 [39]. Despite the thermogenesis-inducing effect of COX-2 [28,34,35,37], depletion of COX-2 in adipocytes did not significantly affect the thermogenic gene expression and energy expenditure under both basal and cold stress conditions [39] (Figure S2). In agreement with this, COX-2 expression levels in adipose tissue are relatively low and do not respond to cold stress [36]. Whereas adipose COX-2 is induced by IF and mediates IF-induced thermogenesis [39]. On the other hand, whether COX-2 plays a role in regulating adipogenesis is still controversial. The pharmaceutical inhibition of COX-2 and antisense COX-2 expression enhanced lipid droplet accumulation during adipogenesis [46,58]. Whereas global deficiency of COX-2 suppressed adipogenic marker expression and decreased fat mass and body mass [29]. In addition, COX-2 has been shown to favor brown or beige adipocyte development [28,59]. The present study used an adipocyte-specific COX-2 KO mouse model and demonstrates that COX-2 in adipocytes plays a negative role in regulating white adipogenesis via PGE2-mediated paracrine mechanisms. It is likely that COX-2/PGE2 signaling has a distinct role in various types of adipogenesis. Rather than PGE2 receptors EP1 and EP2, EP4 mediates the suppressing effect of PGE2 on white adipocyte differentiation by targeting the early stage of differentiation [60] (Figure 4).

In summary, our data show that adipocyte-derived PGE2 serves as a paracrine signal that limits white adipogenesis. PGE2 suppresses adipogenesis through the PKA/PPARγ pathway in preadipocytes. As a result, adipocyte-specific COX-2 deficient mice displayed exacerbated diet-induced adiposity, obesity, and insulin resistance, a phenotype that was reversed by PGE2 administration. Our study uncovers that the COX-2/PGE2 axis in adipocytes is a key regulator of adipose tissue expansion in obesity.

## Data Availability

Not applicable.

## Supplementary Materials

The following supporting information can be downloaded at: https://www.mdpi.com/article/10.3390/cells11111819/s1, Figure S1: COX-2 KO mice displays no differences in food intake and insulin sensitivity after 16 week-HFD feeding and female COX-2 KO mice did not exhibit similar phenotype as males; Figure S2: Deficiency of COX-2 in adipocytes has little effect on energy expenditure under normal chow diet condition; Figure S3: Deficiency of COX-2 in adipocytes improves type 2 inflammation at NCD but not 16-week HFD conditions; Figure S4: PGE2 suppresses PPARγ expression and adipogenesis through PKA signaling; Table S1: List of primer sequences used in real-time PCR.

## Abbreviations

BAT, brown adipose tissue; COX-2, Cyclooxygenase 2; COX-1, Cyclooxygenase 1; gWAT, gonadal WAT; iWAT, inguinal WAT; PG, prostaglandin; PGs, prostaglandins; PGE2, prostaglandin E2; PGI2, prostacyclin; PGD2, prostaglandin D2; PKA, cAMP-dependent protein kinase; WAT, white adipose tissue; PPARγ, Peroxisome Proliferator Activated Receptor γ; PGC1α, peroxisome proliferator-activated receptor gamma coactivator 1-alpha; UCP1, uncoupling protein 1; *C/ebpα*, CCAAT/enhancer-binding protein alpha; IFNγ, interferon γ; TNFα, tumor necrosis factor alpha; Plin1, lipid droplet-associated protein 1; IF, intermittent fasting; H&E staining, hematoxylin and eosin staining.
