# Supplementary material for: COX-2 Deficiency Promotes White Adipogenesis via PGE2-Mediated Paracrine Mechanism and Exacerbates Diet-Induced Obesity
Source: Cells. 2022 Jun 2;11(11):1819. doi: 10.3390/cells11111819 (PMC9180646; doi:10.3390/cells11111819)
Supplement: Supplementary file 1 [file cells-11-01819-s001.zip › cells-1726398-supplementary.pdf]

Supplementary information

Table S1. List of primer sequences used in real-time PCR.

Gene	Forward sequence (5' to 3')	Reverse sequence (5' to 3')
Tubulinβ	CAGGCCGGACAGTGTGGCAAC	GGCTTCATTATAGTACACAGAGATTCG
COX-2	CTCACGAAGGAACTCAGCACT	TAGAATCCAGTCCGGGTACAGT
PPARγ	TGTGGACCTCTCCGTGATGG	GGTTCTACTTTGATCGCACTTTGG
ADPN	GGGTGAGACAGGAGATGTTGGAATG	GCCAGTAAATGTAGAGTCGTTGACG
Plin1	GGGACCTGTGAGTGCTTCC	GTATTGAAGAGCCGGGATCTTTT
Foxp3	CACCTATGCCACCCTTATCCG	CATGCGAGTAAACCAATGGTAGA
GATA-3	TTTACCCTCCGGCTTCATCCTCCT	TGCACCTGATACTTGAGGCACTCT
IL-10	CTTACTGACTGGCATGAGGATCA	GCAGCTCTAGGAGCATGTGG
IFN-γ	ATCTGGAGGAACTGGCAAAA	TTCAAGACTTCAAAGAGTCTGAGGTA

Figure legends:

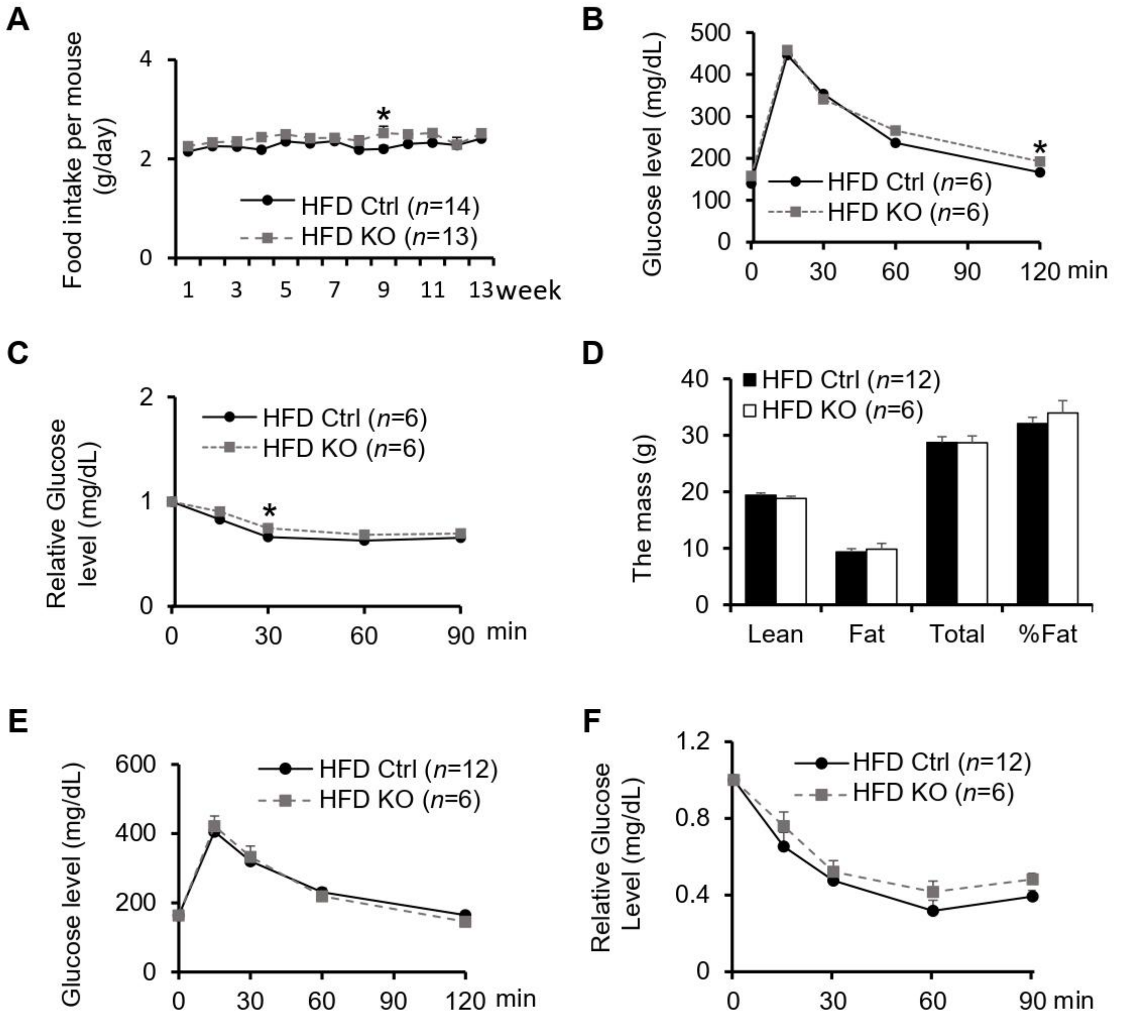

Figure.S1. COX-2 KO mice displays no differences in food intake and insulin sensitivity after 16 week-HFD feeding and female COX-2 KO mice did not exhibit similar phenotype as males.

6-week-old male COX-2 KO and control mice were fed with high fat diet (HFD) for 16 weeks. **A**. No significant difference in food intake between KO and control mice was observed during HFD feeding. Ctrl, control. After 16-week HFD feeding, glucose (**B**) and insulin tolerance (**C**) were slightly worse in COX-2 KO mice compared to control mice. 6-week-old female COX-2 KO and control mice were fed with high fat diet (HFD) for 8 weeks. **D**. There was no significant difference in body mass, fat mass and fat percentage between HFD-fed COX-2 KO and control mice. DEXA scanning was performed to determine the body mass, fat mass and fat percentage. Glucose (**E**) and insulin tolerance (**F**) was little affected by COX-2 KO in female mice under HFD feeding conditions. Data are presented as the mean ±SEM. T-Test was used for the analysis in Figs. S1A and S1D, and ANOVA was used for the analysis of Figs. S1B-S1C and S1E-S1F. *$p<0.05$, HFD control vs. HFD KO.

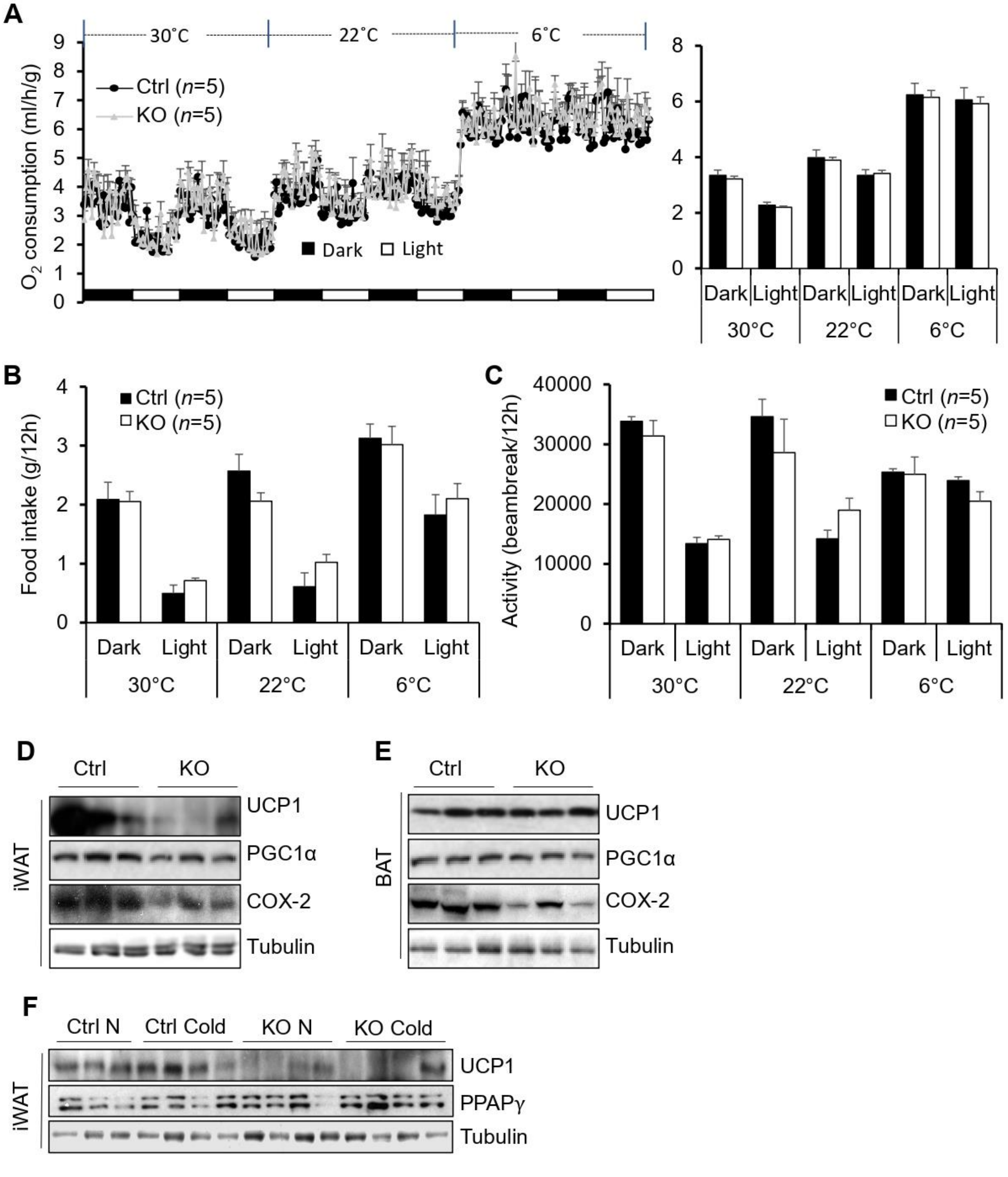

Figure.S2. Deficiency of COX-2 in adipocytes has little effect on energy expenditure under normal chow diet condition.

3-month-old normal chow diet (NC) male mice underwent Promethion Metabolic Phenotyping Systems (Sable Systems International). Mice were individually housed in the metabolic cages coupled with an environmental chamber and stayed at 30˚C, 22˚C, and 6˚C each for two days. **A**. COX-2 KO mice displayed similar O2 consumption level under 30˚C, 22˚C, and 6˚C conditions

as control mice. O_2 consumption was normalized by lean body mass and analyzed by ANOVA. Food intake (**B**) and activity (**C**) were not significantly affected by COX-2 KO compared to control mice. The expression levels of thermogenic markers UCP1 and PGC1α was downregulated in COX-2 KO iWAT (**D**) with little changes in BAT (**E**). **F**. The expression level of UCP1 were induced by cold stress in control mice, while COX-2 had no significant effect on cold-induced UCP1 expression in iWAT. In addition, the protein levels of PPARγ were induced by COX-2 KO in iWAT regardless of cold stress. Data are presented as the mean ±SEM. T-Test was used for the analysis in Figs. S2B and S2C.

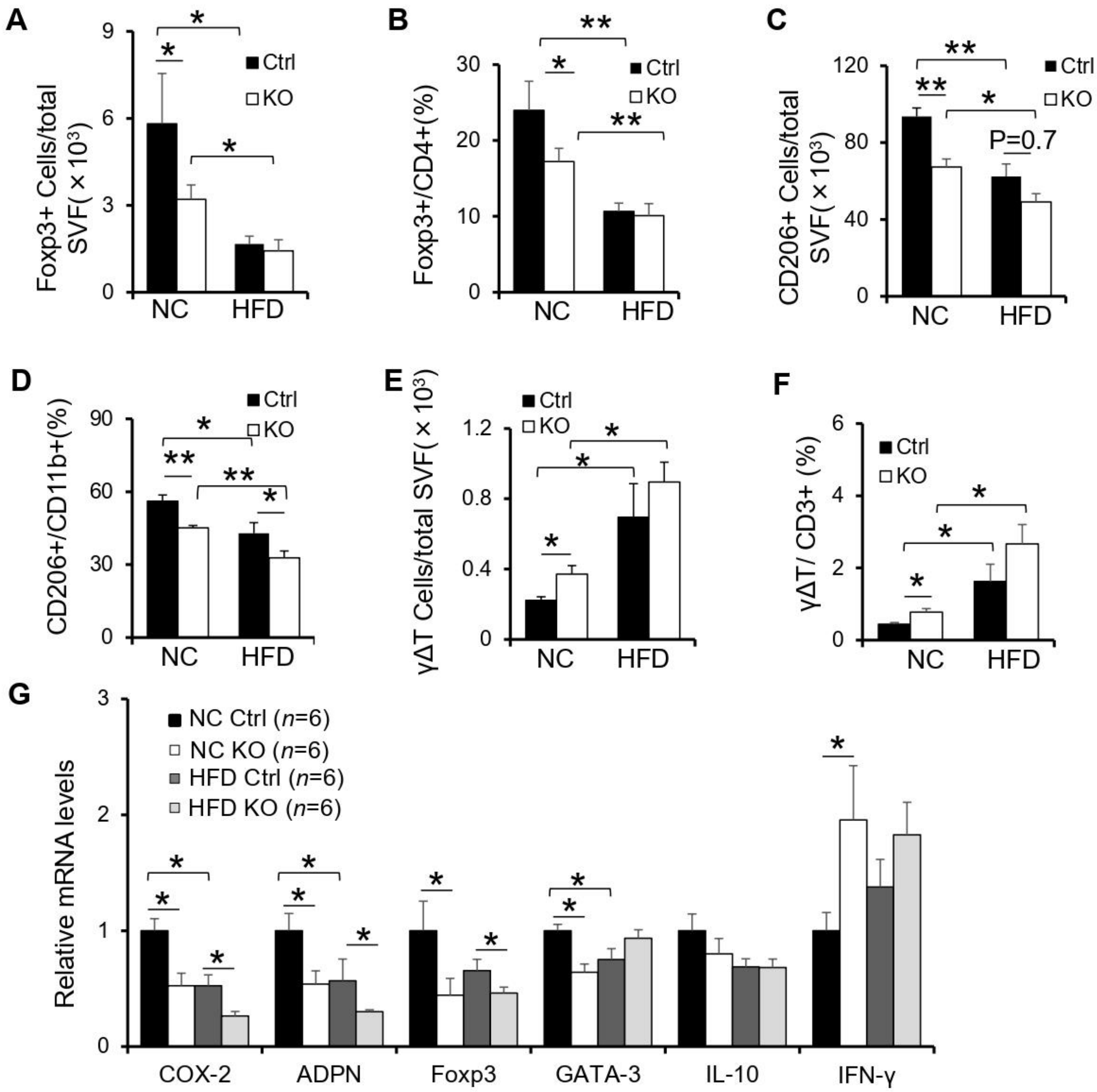

Figure.S3. Deficiency of COX-2 in adipocytes improves type 2 inflammation at NCD but not 16-week HFD conditions.

6-week-old male COX-2 KO and control mice were fed with normal chow diet (NCD) or high fat diet (HFD) for 16 weeks. The gWAT samples were then collected and used for the following studies. The stromal vascular fraction of adipose tissue was used for Flow Cytometry analysis. **A**. The fraction of resident Foxp3$^+$/CD4$^+$ cells was decreased in gWAT of COX-2 KO mice compared to control mice under NCD condition, while this decrease disappeared under HFD condition. *n*=6-14/group. **B**. The percentage of Foxp3+/CD4$^+$ cells in total CD4$^+$ cells was decreased in gWAT of COX-2 KO mice compared to control mice under NCD but not HFD conditions. *n*=6-14/group. COX-2 KO mice displayed reduced CD206$^+$CD11b$^+$ population (**C**) and decreased ratio of CD206$^+$CD11b$^+$ in CD11b$^+$ cells (**D**) in gWAT under NCD condition, and this decrease was

abrogated under HFD condition. *n*=5-11/group. COX-2 deficiency led to an increase in $\gamma\Delta T^{+}CD3^{+}$ cell population (**E**) and in the ratio of $\gamma\Delta T^{+}CD3^{+}$ cell in total CD3+ cells (**F**) in gWAT under NCD condition. *n*=6-14/group. **G**. COX-2 KO downregulated mRNA levels of adiponectin, Foxp3, GATA-3 as well as COX-2 in gWAT under both NCD and HFD conditions, while upregulating levels of IFN-γ in gWAT under NCD conditions despite no significant effect on IL-10. Data are presented as the mean ±SEM. T-Test was used for the analysis in Figs. S3A to S3G. *p<0.05, **p <0.01.

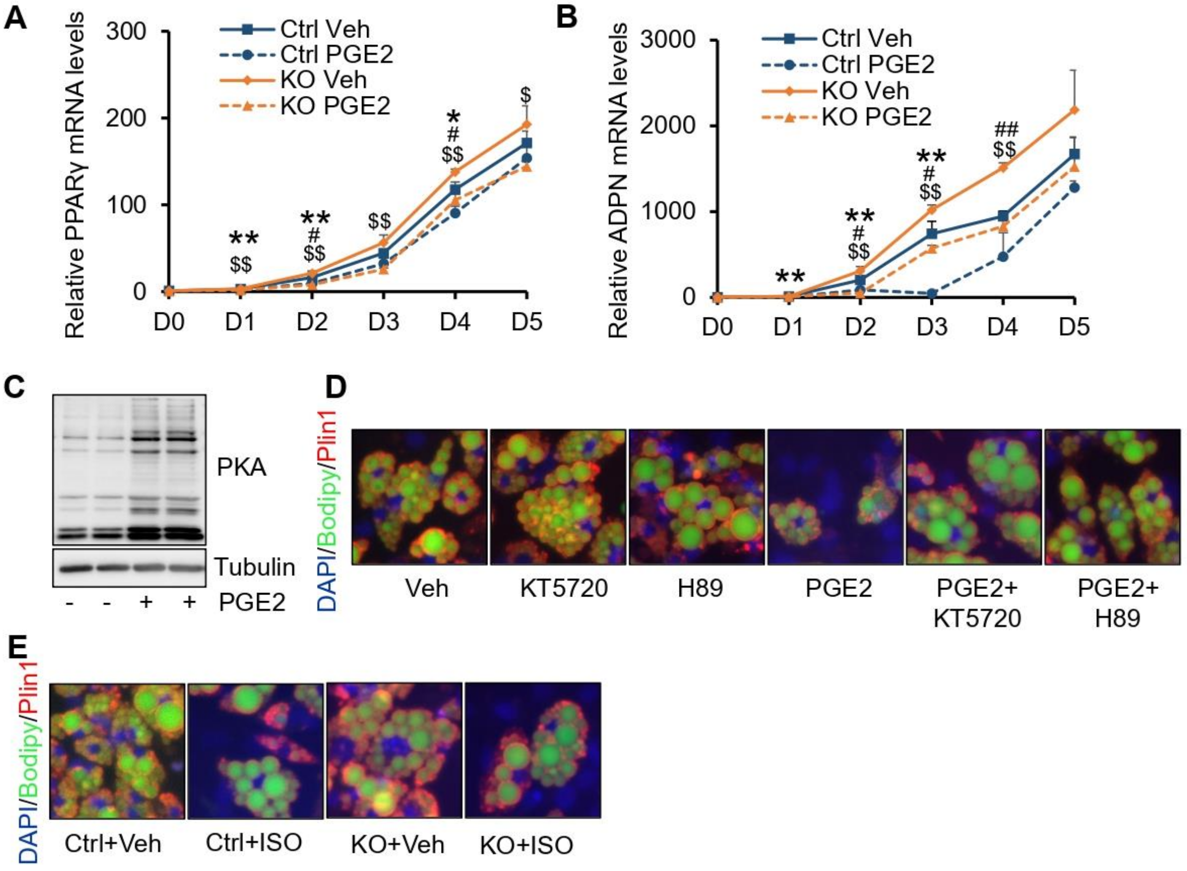

Figure.S4. PGE2 suppresses PPARγ expression and adipogenesis through PKA signaling.

Primary stromal vascular fractions from inguinal fat depots of 3-week-old COX-2 KO and Ctrl mice were isolated, cultured and differentiated into adipocytes. COX-2 KO induced the mRNA levels of PPARγ (**A**) and adiponectin (ADPN) (**B**), and this induction was suppressed by PGE2 treatment. During the differentiation, the cells were harvested on different days from D0 to D5 and analyzed by Real-Time PCR. *p<0.05, **p<0.01, Ctrl Veh vs. Ctrl PGE2; #p<0.05, ##p<0.01, Ctrl Veh vs. KO Veh; $$p$<0.05, $$$p$<0.01, KO Veh vs. KO PGE2. n=4/group. **C**. PKA substrates were activated by PGE2 treatment for 2 hrs. **D**. PGE2 suppressed white adipogenesis and this suppression were restored by inhibition of PKA with its specific inhibitor KT5720 or H89 indicated by fluorescent staining of Bodipy, Plin1 and DAPI. **E**. Activation of PKA with isoproterenol suppressed COX-2 deficiency-induced white adipogenesis as presented by the fluorescent staining of Bodipy, Plin1 and DAPI. Figs. S4D-S4E are the representative data from three independent experiments. Data are presented as the mean ±SEM. ANOVA was used for the analysis of Figs. S4A and S4B.
